# Exploratory analysis to identify the best antigen and the best immune biomarkers to study SARS-CoV-2 infection

**DOI:** 10.1186/s12967-021-02938-8

**Published:** 2021-06-26

**Authors:** Elisa Petruccioli, Saeid Najafi Fard, Assunta Navarra, Linda Petrone, Valentina Vanini, Gilda Cuzzi, Gina Gualano, Luca Pierelli, Antonio Bertoletti, Emanuele Nicastri, Fabrizio Palmieri, Giuseppe Ippolito, Delia Goletti

**Affiliations:** 1 Translational Research Unit, National Institute for Infectious Diseases Lazzaro Spallanzani-IRCCS, Rome, Italy; 2 Clinical Epidemiology Unit, National Institute for Infectious Disease Lazzaro Spallanzani-IRCCS, Rome, Italy; 3 UOS Professioni Sanitarie Tecniche National Institute for Infectious Diseases Lazzaro Spallanzani-IRCCS, Rome, Italy; 4 Clinical Division of Infectious Diseases, National Institute for Infectious Diseases Lazzaro Spallanzani-IRCCS, Rome, Italy; 5 UOC Transfusion Medicine and Stem Cell Unit, San Camillo Forlanini Hospital, Rome, Italy; 6 Programme in Emerging Infectious Diseases, Duke-National University of Singapore Medical School, Singapore, Singapore; 7 Scientific Direction, National Institute for Infectious Disease “Lazzaro Spallanzani”-IRCCS, Rome, Italy

**Keywords:** SARS-CoV-2, COVID-19, Biomarkers, T-cell, Immunity, IP-10, Whole-blood, Immune response, Spike, IFN-γ

## Abstract

**Background:**

Recent studies proposed the whole-blood based IFN-γ-release assay to study the antigen-specific SARS-CoV-2 response. Since the early prediction of disease progression could help to assess the optimal treatment strategies, an integrated knowledge of T-cell and antibody response lays the foundation to develop biomarkers monitoring the COVID-19. Whole-blood-platform tests based on the immune response detection to SARS-CoV2 peptides is a new approach to discriminate COVID-19-patients from uninfected-individuals and to evaluate the immunogenicity of vaccine candidates, monitoring the immune response in vaccine trial and supporting the serological diagnostics results. Here, we aimed to identify in the whole-blood-platform the best immunogenic viral antigen and the best immune biomarker to identify COVID-19-patients.

**Methods:**

Whole-blood was overnight-stimulated with SARS-CoV-2 peptide pools of nucleoprotein-(NP) Membrane-, ORF3a- and Spike-protein. We evaluated: IL-1β, IL-1Ra, IL-2, IL-4, IL-5, IL-6, IL-7, IL-8, IL-9, IL-10, IL-12p70, IL-13, IL- 15, IL-17A, eotaxin, FGF, G-CSF, GM-CSF, IFN-γ, IP-10, MCP-1, MIP-1α, MIP-1β, PDGF, RANTES, TNF-α, VEGF. By a sparse partial least squares discriminant analysis we identified the most important soluble factors discriminating COVID-19- from NO-COVID-19-individuals*.*

**Results:**

We identified a COVID-19 signature based on six immune factors: IFN-γ, IP-10 and IL-2 induced by Spike; RANTES and IP-10 induced by NP and IL-2 induced by ORF3a. We demonstrated that the test based on IP-10 induced by Spike had the highest AUC (0.85, p  <  0.0001) and that the clinical characteristics of the COVID-19-patients did not affect IP-10 production. Finally, we validated the use of IP-10 as biomarker for SARS-CoV2 infection in two additional COVID-19-patients cohorts.

**Conclusions:**

We set-up a whole-blood assay identifying the best antigen to induce a T-cell response and the best biomarkers for SARS-CoV-2 infection evaluating patients with acute COVID-19 and recovered patients. We focused on IP-10, already described as a potential biomarker for other infectious disease such as tuberculosis and HCV. An additional application of this test is the evaluation of immune response in SARS-CoV-2 vaccine trials: the IP-10 detection may define the immunogenicity of a Spike-based vaccine, whereas the immune response to the virus may be evaluated detecting other soluble factors induced by other viral-antigens.

## Introduction

COronaVIrus Disease-2019 (COVID-19) pandemic is caused by the novel coronavirus designated as severe acute respiratory syndrome coronavirus (SARS-CoV)-2 [1] belonging to β-Coronovavirus genus. Its genome contains 14 open reading frames (ORFs) and encodes 27 different proteins, including spike (S), envelope (E), membrane (M) and nucleocapsid (NP) proteins [2]. The majority of people with COVID-19 develop mild (40%) or moderate (40%) symptoms, 15–20% develop a severe disease needing oxygen support and 5% have a critical disease with complications such as respiratory failure, acute respiratory distress syndrome (ARDS), sepsis and septic shock, thromboembolism, and/or multi-organ failure [3–5]. SARS-CoV-2 infection induces an immune response in the host characterized in severe COVID-19 cases by a decrease of lymphocytes number and a great increase of cytokines [6]. Currently, the mechanisms that lead to disease exacerbation remains largely undetermined. Thus, there is an urgent need to improve our understanding of the immunology of this disease to find correlate of protection or to monitor the course of the infection.

Several reports described different immune profiles of COVID-19-patients according to the diseases [7–15]. SARS-CoV-2 infection decreases the lymphocytes number and increases cytokines release in severe COVID-19-cases [14]. A significant increase of pro-inflammatory or anti-inflammatory cytokines, including T helper (Th) type-1 and type-2 cytokines and chemokines was described [10, 12, 16, 17], interleukin (IL)-1β, IL-6, IL-8, and Interferon (IFN)-γ-inducible protein (IP-10) were associated with severe or fatal course of disease [7–9]. Four immune signatures, constituted by growth factors, Th1-, Th2-, Th3-cytokines and chemokines, were correlated with distinct disease courses [9]. In acute and convalescent subjects, a coordinated immune response of T-cells and antibodies was associated with milder disease [13]. The importance of T-cell response against β-coronavirus infections has been underlined by a study on patients recovered from SARS, demonstrating the persistence of long-lasting memory T-cells reactive to SARS-CoV stimulation, years after the SARS-outbreak in 2003 [18, 19]. Recent studies highlighted the use of the whole-blood based IFN-γ released assay as a promising approach to study the antigen-specific SARS-CoV-2 response [10–12, 20, 21]. The use of a whole-blood-platform with SARS-CoV2 peptides to discriminate COVID-19-patients and uninfected-individuals [10, 20, 22], is a new potential approach to study the immunogenicity of vaccine candidate, to monitor the immune response in vaccine trial and to support the serological diagnostics.

In this study, we analyzed in a whole-blood-cytokine platform, the best approach to evaluate the SARS-CoV-2-T-cell response to the structural (N, S and M) [19] and accessory protein (ORF3a) [23, 24] of SARS-CoV-2. We aimed to identify (i) the best antigen to induce the SARS-CoV-2 specific T-cell response; (ii) the best subset of biomarkers to identify COVID-19-patients.

## Results

### Identification of plasma biomarkers for distinguishing COVID-19 from NO-COVID-19-individuals

Demographical and clinical information of the enrolled subjects are shown in Table 1. We stimulated the whole-blood of with SARS-CoV-2-specific peptide pools of NP (NP Pool1 and NP Pool2), Membrane, ORF3a, and Spike. Then, we evaluated by luminex the plasma level of 27 analytes. Among the different stimuli, the Spike and NP Pool1 peptides, belonging both to SARS-CoV-2 structural proteins, were the most recognized antigens by COVID-19-patients (Table 2). Spike peptide pool was the most immunogenic stimulus, modulating the highest number of cytokines, chemokines and growth factors (Table 2).

**Table 1:** Demographical and clinical characteristics of the enrolled subjects

	COVID-19	NO-COVID-19	p value
N (%)	23 (56.1)	18 (43.9)	–
Age median (IQR)	45 (35–57)	48.5 (33.25–59.5)	0.73*
Male N (%)	19 (82.6)	11 (61.1)	0.123^§^
Origin N (%)			0.014^§^
West Europe	11 (48)	15 (83)	
East Europe	0 (0)	2 (11)	
Asia	9 (39)	0 (0)	
Africa	1 (4.3)	0 (0)	
South America	2 (8.7)	1 (6)	
Swab positive results N (%)	23 (100)	0 (0)	
Serology results IgM. N (%)			
IgM +	12 (52.2)	0 (0)	
IgM −	11 (47.8)	18 (100)	
Serology results IgG. N (%)			
IgG +	13 (56.5)	0 (0)	
IgG −	8 (34.8)	18 (100)	
IgG doubtful	2 (8.7)	0 (0)	
Severity N (%)^a^			
Asymptomatic	2 (8.7)	–	
Mild	3 (13)	–	
Moderate	11 (48)	–	
Severe	5 (21.7)	–	
Critical	2 (8.6)	–	
Cortisone N (%)	6 (26)	–	
Severity of patients taking cortisone N (%)			
Asymptomatic	0 (0)	–	
Mild	0 (0)	–	
Moderate	2 (33)	–	
Severe	3 (50)	–	
Critical	1 (17)	–	

*COVID-19* Coronavirus Disease 19; *N* number

^a^WHO criteria (1)

*Mann Whitney test

^§^Chi-square test

**Table 2:** Cytokines, chemokines and grow factors significantly modulated in COVID-19 and NO-COVID-19 individuals

	Main source	Analytes	NP Pool1			NP Pool2			Spike			ORF3a			Membrane		
			COVID-19	NO-COVID-19	p*	COVID-19	NO-COVID-19	p*	COVID-19	NO-COVID-19	p*	COVID-19	NO-COVID-19	p*	COVID-19	NO-COVID-19	p*
			Median (IQR)	Median (IQR)		Median (IQR)	Median (IQR)		Median (IQR)	Median (IQR)		Median (IQR)	Median (IQR)		Median (IQR)	Median (IQR)	
Inflammatory cytokines/chemokines	Macrophages	IL-6	–	–	–	–	–	–	51.56 (22.2–98.48)	8.98 (6.09–27.1)	0.008	–	–	–	–	–	–
	Macrophages	TNF-α	28.84 (15.72–40.99)	4.5 (0.0–21.44)	0.009	26.72 (7.04–55.16)	4.84 (0.0–32.89)	0.048	33.88 (16.32–70.8)	14.08 (5.71–23.81)	0.0067	–	–	–	–	–	–
	Th1	IFN-γ	–	–	–	–	–	–	131.2 (101.2–243)	48.2 (25.04–118.6)	0.017	–	–	–	–	–	–
	Th17	IL-17	–	–	–	–	–	–	9.4 (4.44–15.44)	2.88 (1.06–6.76)	0.017	–	–	–	–	–	–
	Monocytes	IP-10	546.2 (0.0–1751)	26.78 (0.0–279.1)	0.025	1349 (0.0–5328)	34.56 (0.0–123.6)	0.028	1108 (289.2–3145)	19.36 (0.0–164.7)	< 0.0001	–	–	–	–	–	–
	Fibroblasts																
	Endothelial cells																
	Monocytes	MCP-1	2548 (1526–3125)	615.7 (138.4–1941)	0.043	–	–	–	2679 (1350–4600)	712.8 (220–1295)	0.013	7754 (3901–10,540)	11,377 (8489–17,050)	0.01	7352 (2138–11,063)	12,841 (7076–13,557)	0.02
	Macrophages																
	Monocytes	MIP-1α	16.84 (6.68–35.2)	3.18 (1.57–11.18)	0.0024	–	–	–	17.6 (10.48–56.12)	7.2 (2.64–15.29)	0.0137	–	–	–	–	–	–
	Macrophages																
	Monocytes	MIP-1β	250.2 (128–335.9)	51.66 (4.86–96.05)	0.0012	–	–	–	382 (177.8–922)	192.8 (65.69–323)	0.014	–	–	–	–	–	–
	Macrophages																
	Platelets. macrophages	RANTES	540.3 (259.8–1013)	0 (0.0–229.6)	0.001	–	–	–	590.8 (407.2–877)	303.8 (0.0–690.9)	0.049	–	–	–	–	–	–
Anti-inflammatory cytokines	Th2	IL-4	0.92 (0.68–1.68)	0.32 (0.12–0.94)	0.0087	–	–	–	1.24 (0.64–2.64)	0.38 (0.07–1.44)	0.015	–	–	–	–	–	–
	Th2	IL-10	1.84 (0.96–6.04)	0.96 (0.0–2.13)	0.046	–	–	–				–	–	–	–	–	–
	Treg																
	Th2	IL-13	–	–	–	–	–	–	0.6 (0.28–2.44)	0.08 (0.0–0.39)	0.043	–	–	–	–	–	–
Growth factors	Th1	IL-2	10.44 (2.88–32.84)	1.78 (0.75–5.24)	0.0023	21.72 (6.12–103)	3.1 (0.3–10.89)	0.0065	32.84 (9.44–100.5)	2.34 (1.13–9.78)	0.0018	59.28 (30.56–142)	31.14 (20.52–62.02)	0.039	64.16 (28.32–213.8)	30.28 (12–62.14)	0.03
	Th9	IL-9	–	–	–	–	–	–	26.24 (0.76–32.2)	6.02 (0.0–24.6)	0.026	–	–	–	–	–	–
	Stromal cells. Macrophages	FGF	–	–	–	–	–	–	25.76 (13.88–40.24)	10.62 (6.9–19.22)	0.017	–	–	–	–	–	–

*COVID-19* CoronaVIrus Disease 19; *N* Number; *IL* interleukin; FGF: fibroblast growth factor, IFN: interferon, IP: IFN-γ-induced protein, MCP: monocyte chemoattractant protein, MIP: macrophage inflammatory protein, RANTES: regulated on activation, normal T cell expressed and secreted, TNF: tumour necrosis factor

^*^Mann Whitney test

**Fig. 1:**
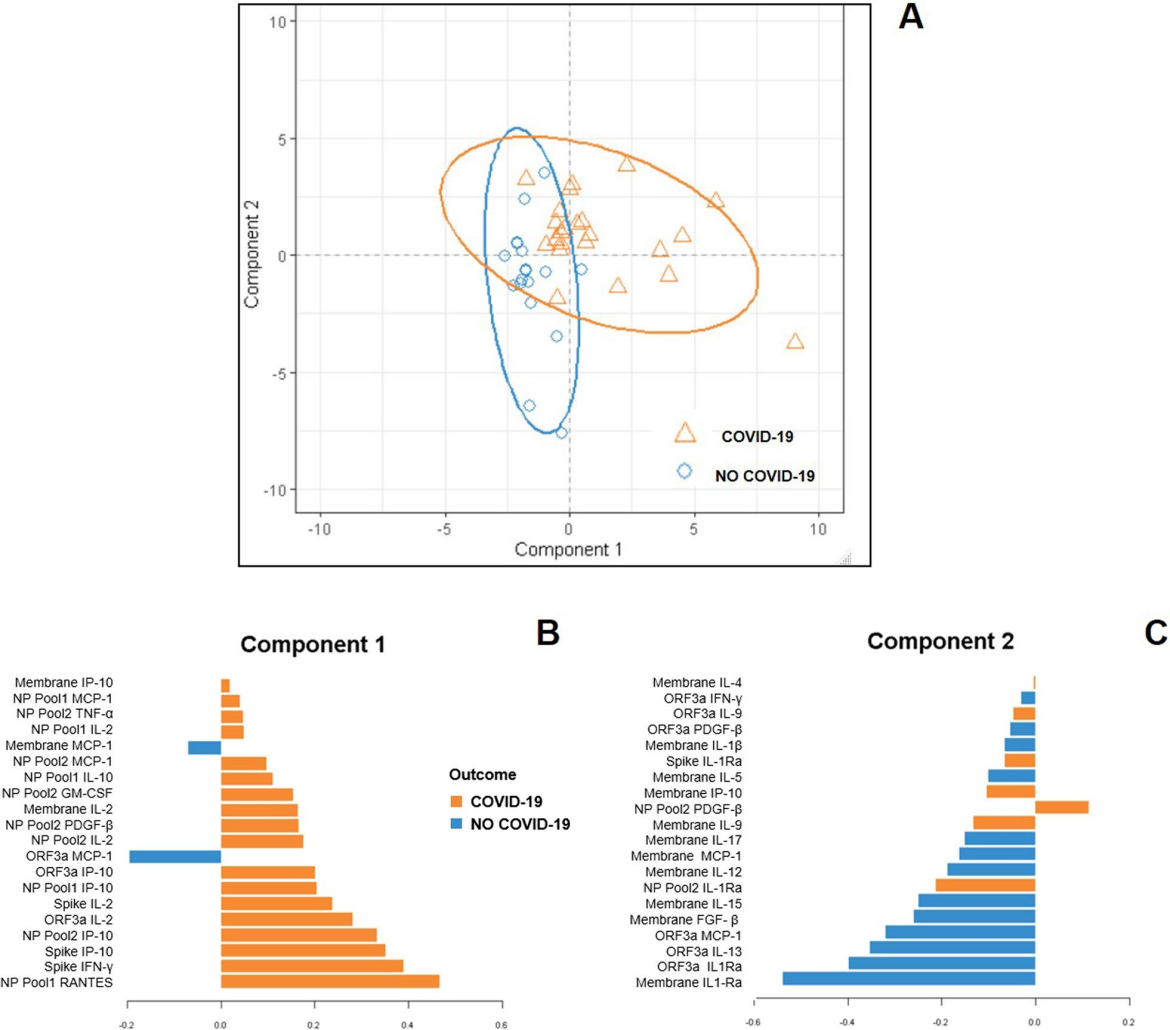
Sparse partial least squares discriminant analysis (sPLS-DA) on luminex data-set of COVID-19 and NO-COVID-19 subjects. The different soluble factors have been measured by luminex assay in plasma collected after stimulating whole-blood with different SARS-CoV-2 peptides (Spike, NP Pool1, NP Pool2, Membrane and ORF3a). **A** The samples are projected in the space spanned by the first two components with 95% confidence level ellipse plots. Colours and symbols indicate the class of each sample (orange triangle COVID-19 patients, blue circles NO-COVID-19 individuals). **B**, **C** Selected immune responses distinguish COVID-19 and NO-COVID-19 individuals over all the evaluated antigens stimulation and distinct immune response detected. The graphs represent the loading weights of the selected variables on each component (20 soluble factors for each component). The variables contribution ranked from the bottom, the most important, to the top. The colors indicate the class for which the selected variable has a maximal mean value: orange COVID-19 patients, blue NO-COVID-19

**Fig. 2:**
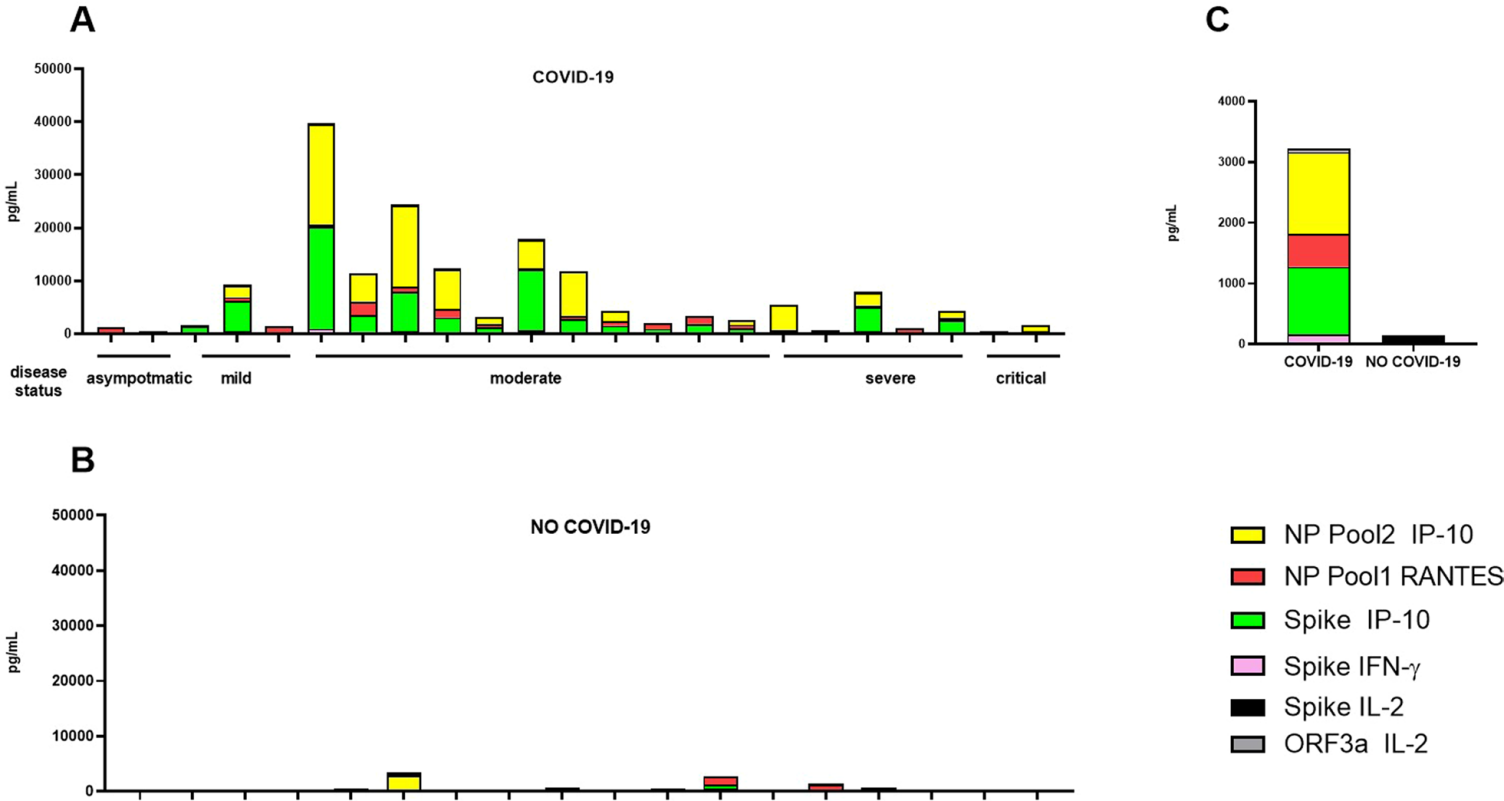
COVID-19 signature based on six selected immune factors. The graphs represent the proportion of immune factors secreted in response to SARS-CoV-2 peptides stimulation within the six variables immune signature associated to COVID-19: RANTES induced by NP Pool1; IFN-γ by Spike; IP-10 by Spike; IP-10 by NP Pool2; IL-2 by Spike, IL-2 by ORF3a. The different immune factors were measured by luminex assay in plasma collected after stimulating whole-blood with the different antigens. **A** Proportion of selected immune factors in COVID-19 patients. **B** Proportion of selected immune factors in NO-COVID-19 subjects. **C** Median proportion of selected immune factors in COVID-19 and NO-COVID-19 subjects. *NP* nucleoprotein, *IL* interleukin; *IP* interferon-γ inducible protein; *IFN* interferon; *RANTES* regulated on activation, normal T cell expressed and secreted

Applying a supervised sPLS-DA we aimed to identify the most important soluble factors, analyzing at the same time the luminex results and the different SARS-CoV-2-peptides pool stimulations (Fig. 1). Although the difference was not fully discriminative, the distribution of COVID-19 and NO-COVID-19-subjects in the space were quite separated (Fig. 1A). Evaluating the loading weights of each selected variable on each component, the mean level of production for the most important selected variables was maximal in COVID-19-patients within the component 1 (Fig. 1B), whereas the mean level of production was maximal in the NO-COVID-19 within the component 2 (Fig. 1C). Overall, the accuracy of the classification was high for both components (>  92%) (data not shown). Since the component 1 was represented mainly by factors upregulated in COVID-19-patients, we focused on this component. Then, we identified the six variables with the highest weight in the construction of component 1 (Fig. 1B–C): IL-2, IFN-γ and IP-10 induced by Spike, regulated on activation, normal T cell expressed and secreted (RANTES) induced by NP Pool1, IP-10 induced by NP Pool2, and IL-2 induced by ORF3a stimulation (hereafter referred as Spike IL-2, Spike IFN-γ, Spike IP-10, NP Pool1 RANTES, NP Pool2 IP-10, and ORF3a IL-2). Next, we evaluated, within the six variables signature associated to COVID-19, the proportion of response to each stimulus: IP-10 proportions induced by Spike and NP Pool2 were the most represented in COVID-19-patients (Fig. 2).

### Comparison of AUC of the six immune factors

**Fig. 3:**
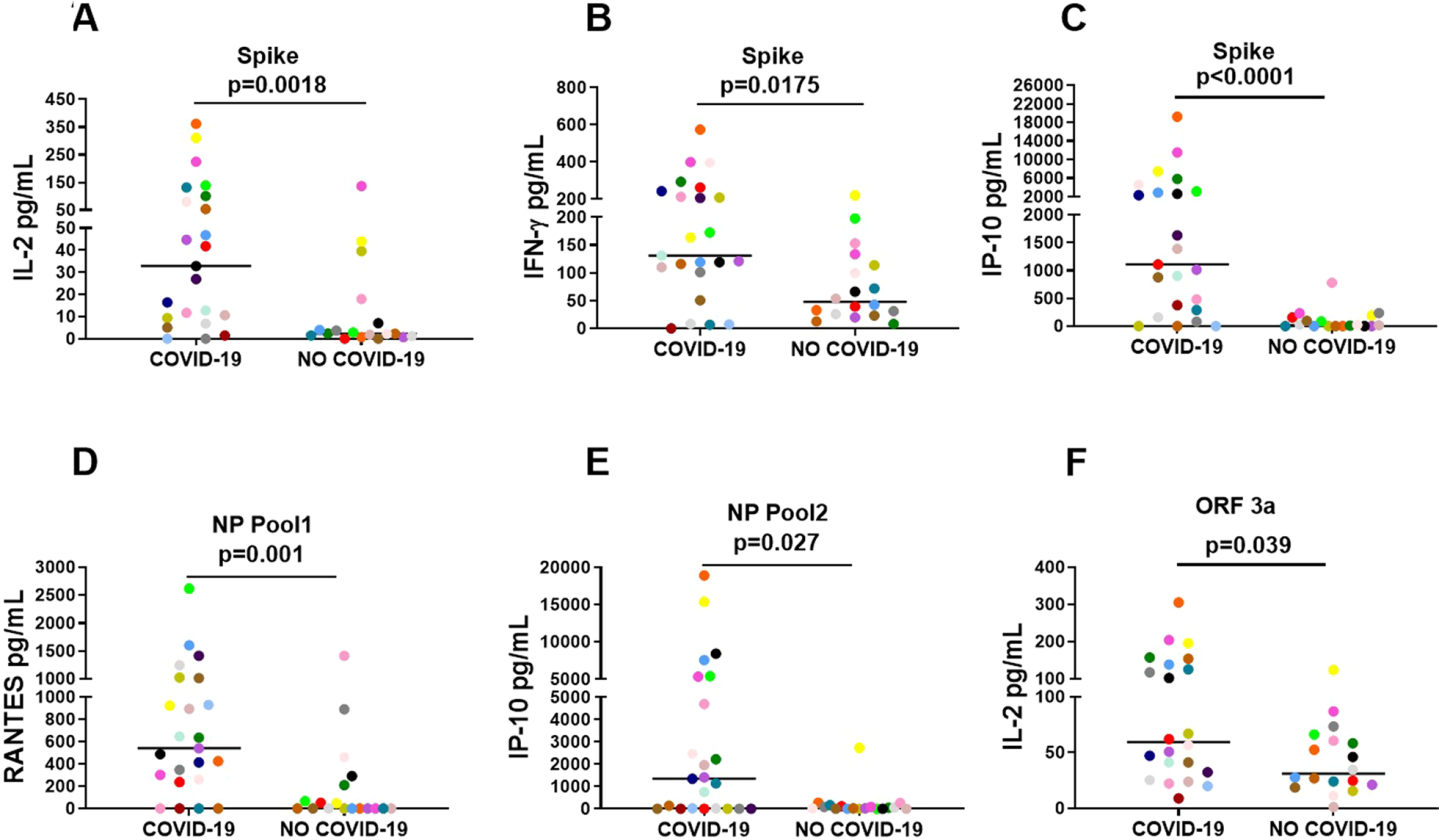
Increased antigen-specific response to selected SARS-CoV-2 antigens in whole-blood is associated with COVID-19. **A** IL-2 production induced by Spike stimulation. **B** IFN-γ production induced by Spike stimulation. **C** IP-10 production induced by Spike stimulation. **D** RANTES production induced by NP Pool1 stimulation. **E** IP-10 production induced by NP Pool2 stimulation. **F** IL-2 production induced by ORF3a stimulation. The different immune factors were measured by luminex assay in plasma collected after stimulating whole-blood with the different antigens. The horizontal lines represent the median; statistical analysis was performed using the Mann–Whitney test, and p value was considered significant when  ≤  0.05. *NP* nucleoprotein, *IL* interleukin; *IP* interferon-γ inducible protein; *IFN* interferon, *RANTES* regulated on activation, normal T cell expressed and secreted

**Fig. 4:**
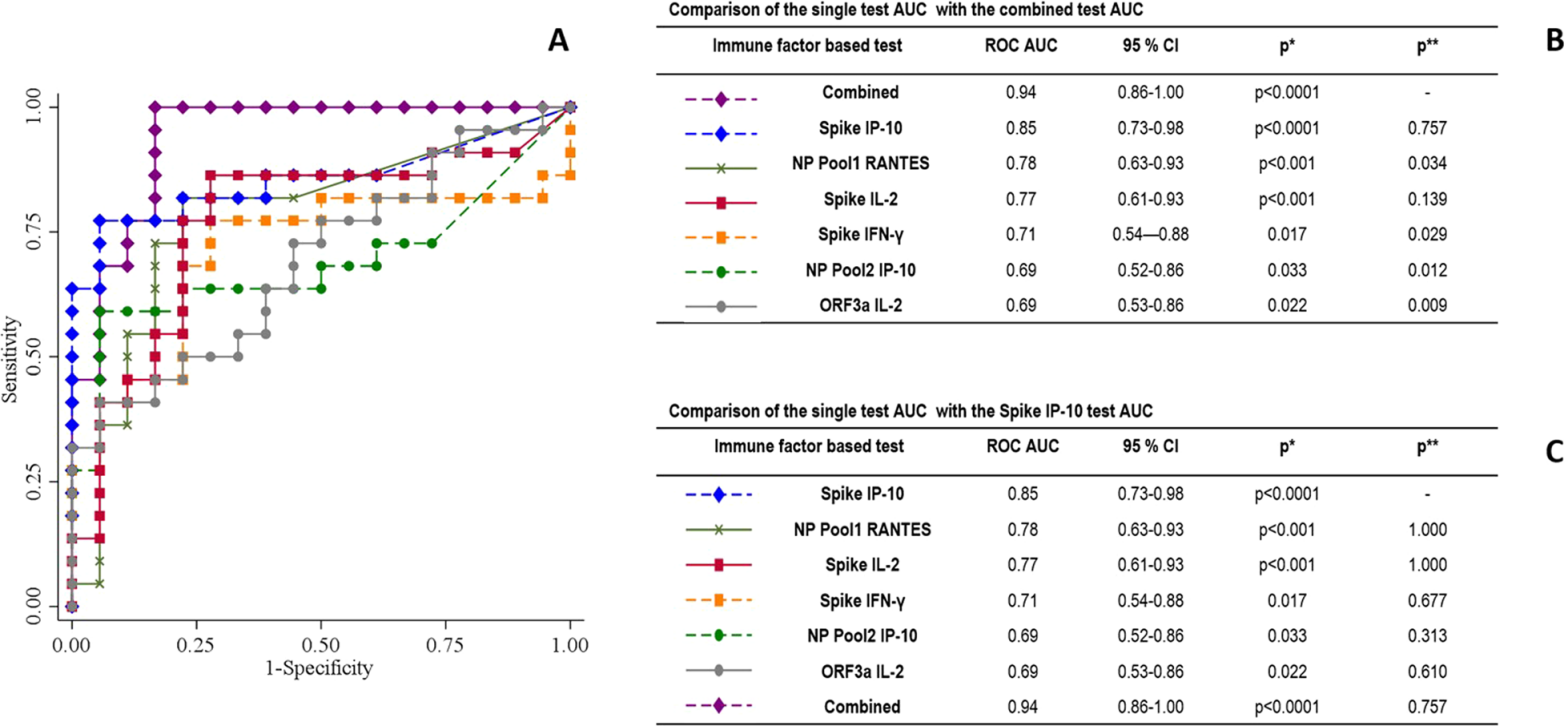
Comparison of the AUC resulting from the SARS-CoV-2-specific responses. **A** The graph shows the AUC of seven different immune responses based on RANTES induced by NP Pool1; IFN-γ by Spike; IP-10 by Spike; IP-10 by NP Pool2; IL-2 by Spike, IL-2 by ORF3a, a combination of all above cited tests (combined test). Since one observation related to IL-2 induced by ORF3a is missing, the AUC comparison has been performed on 40 patients instead of 41. **B** Comparison of the single test AUCs with the combined test: *p values referred to correspondent ROC; **comparison of AUCs of NP Pool 1 RANTES, Spike IFN-γ, Spike IP-10, NP Pool 2 IP-10, Spike IL-2, ORF3a IL-2 with the AUC of combined test. **C** Comparison of the single test AUCs with IP-10 induced by Spike AUC: *p values referred to correspondent ROC; **comparison of AUCs of NP Pool 1 RANTES, Spike IFN-γ, NP Pool 2 IP-10, Spike IL-2, ORF3a IL-2, combined test, with the AUC of Spike IP-10. *IL* interleukin; *IP* interferon-γ inducible protein; *IFN* interferon; *RANTES* regulated on activation, normal T cell expressed and secreted, *NP* nucleoprotein, *CI* confidence interval; *AUC* area under the curve

The selected six immune factors of component 1, as expected, had significant quantitative higher levels in COVID-19 compared to controls for: IL-2, IFN-γ, IP-10 induced by Spike (p  =  0.0018; p  =  0.0175; p  <  0.0001; respectively), NP Pool1 RANTES (p  =  0.001), NP Pool2 IP-10 (p  =  0.027) and ORF3a IL-2 (p  =  0.039) (Fig. 3; Table 2). ROC curve analysis of these factors showed that the highest AUC was related to IP-10 Spike (AUC 0.85; p  <  0.0001; Fig. 4).

Then, we generated a combined-test based on the six immune factors previously selected (Fig. 4). The combined-test showed a significantly further increase of AUC (AUC 0.94; p  <  0.0001) compared to the AUCs of the other single tests except for IP-10 and IL-2 induced by Spike (Fig. 4B). Since IP-10 Spike test showed the highest AUC, we compared it with all the other AUCs and we did not find any significant differences among the different tests (Fig. 4C).

### Impact of the clinical characteristics of patients on the COVID-19 signature

We investigated if any clinical characteristic of COVID-19-patients had an impact on the level of the six selected variables (Table 3). We found that age (p  =  0.001), cortisone (p  =  0.042) and severity of the disease (p  =  0.015) had a significant impact on NP Pool1 RANTES. NP Pool2 IP-10 was modulated by symptoms (p  =  0.036), IgM index (p  =  0.003) and IgM score (p  =  0.017). Finally, ORF3a IL-2 was modulated, by the number of days from the symptoms onset (p  <  0.0001) and IgM index (p  =  0.038). Similarly, Spike IL-2 was modulated by number of days from the symptoms onset (p  =  0.001), IgM index (p  =  0.028) and IgM score (p  =  0.036).

**Table 3:** Impact of the characteristics of COVID-19 patients on the six selected immune factors (IL-2. IFN-γ. IP-10 induced all by Spike; RANTES induced by NP Pool1; IP-10 induced by NP Pool2; IL-2 induced by ORF3a)

Characteristics	NP Pool1 RANTES			Spike INF-γ			Spike IP-10			NP Pool2 IP-10			ORF3a IL-2			Spike IL-2		
	Median (IQR)	r_s_	p	Median (IQR)	r_s_	p	Median (IQR)	r_s_	p	Median (IQR)	r_s_	p	Median (IQR)	r_s_	p	Median (IQR)	r_s_	p
Gender																		
Male	540 (302–928)	Na	0.935	164 (110–262)	Na	0.194	1390 (375–4608)	Na	0.33	1349 (0–5328)	Na	0.513	59 (41–153)	Na	0.67	33 (9–100)	Na	0.935
Female	622 (0–1422)			64 (7–166)			385 (224–1677)			2919 (580–6123)			76 (24–132)			29 (9–89)		
Age	Na	− 0.65	**0.001**	Na	0.03	0.893	Na	− 0.14	0.513	Na	− 0.06	0.792	Na	− 0.15	0.519	Na	0	0.989
Cortisone																		
No	894 (302–1025)	Na	**0.042**	131 (51–208)	Na	0.484	1107 (375–3145)	Na	0.779	1136 (0–2464)	Na	0.34	53 (29–132)	Na	0.376	42 (9–100)	Na	0.916
Yes	380 (0–424)			166 (116–243)			1394 (81–2629)			3025 (140–8414)			109 (47–153)			25 (12–54)		
Sympotms																		
No	237 (0–1025)	Na	0.391	208 (0–262)	Na	0.92	375 (0–1107)	Na	0.191	0 (0–0)	Na	**0.036**	62 (9–67)	Na	0.427	9 (1–42)	Na	0.159
Yes	588 (302–928)			147 (110–243)			1510 (289–4608)			1684 (140–5328)			56 (32–153)			46 (12–132)		
Days from onset sympotms	Na	− 0.21	0.405	Na	0.2	0.416	Na	0.35	0.15	Na	0.41	0.093	Na	0.8	**0**	Na	0.72	**0.001**
Severity of disease																		
asy/mild/	770 (456–1135)	Na	**0.015**	147 (114–235)	Na	0.548	1510 (890–4488)	Na	0.061	1684 (0–6464)	Na	0.567	62 (32–156)	Na	0.647	37 (10–120)	Na	0.462
mod																		
sev/crit	260 (0–414)			116 (7–243)			289 (0–2307)			1136 (18–2464)			56 (22–125)			16 (0–80)		
Lymphocytes (× 10^3^)																		
Percentage	Na	0.43	**0.042**	Na	0.46	**0.026**	Na	0.41	**0.052**	Na	0.06	0.772	Na	0.23	0.299	Na	0.22	0.309
Number	Na	0.28	0.204	Na	0.11	0.631	Na	0.1	0.653	Na	− 0.05	0.83	Na	0.08	0.727	Na	− 0.08	0.727
Serology																		
IgG index	Na	− 0.12	0.594	Na	− 0.12	0.596	Na	− 0.15	0.499	Na	0.17	0.433	Na	0.19	0.385	Na	0.24	0.271
IgG score																		
Negative	491 (281–1153)	Na	0.975	162 (106–345)	Na	0.917	2251 (882–5219)	Na	0.386	2091 (0–3896)	Na	0.823	87 (28–147)	Na	0.384	37 (6–90)	Na	0.351
Positive	540 (237–1013)			131 (116–208)			902 (289–2629)			1136 (26–5385)			64 (41–139)			42 (12–132)		
Doubtful	671 (414–928)			125 (7–243)			1153 (0–2307)			683 (18–1349)			33 (20–47)			8 (0–16)		
IgM index	Na	0.13	0.563		0.21	0.333	Na	0.29	0.157	Na	0.59	**0.003**	Na	0.45	**0.038**	Na	0.46	**0.028**
IgM score																		
Negative	414 (260–1013)	Na	0.666	116 (51–243)	Na	0.538	877 (0–2307)	Na	0.157	18 (0–1960)	Na	**0.017**	47 (24–117)	Na	0.2	11 (1–54)	Na	**0.036**
Positive	588 (331–1084)			147 (119–237)			1868 (692–4488)			3462 (946–7979)			102 (41–157)			46 (23–135)		

Bold values indicate *p* values<0.05

Mann-Whitney or Kruskall-Wallis Test for categorical variables; Spearman’s correlation for continuous variables

*r*_*s*_ : Sperman’s correlation coefficient; *Asy/mild/mod:* asymptomatic/mild/moderate; Sev/crit: severe/critical; *Na:* not applicable

Differently, Spike IFN-γ and Spike IP-10 were not significantly modulated by any of the clinical characteristics considered.

### Evaluation of IP-10 in different cohorts of COVID-19-patients

**Fig. 5:**
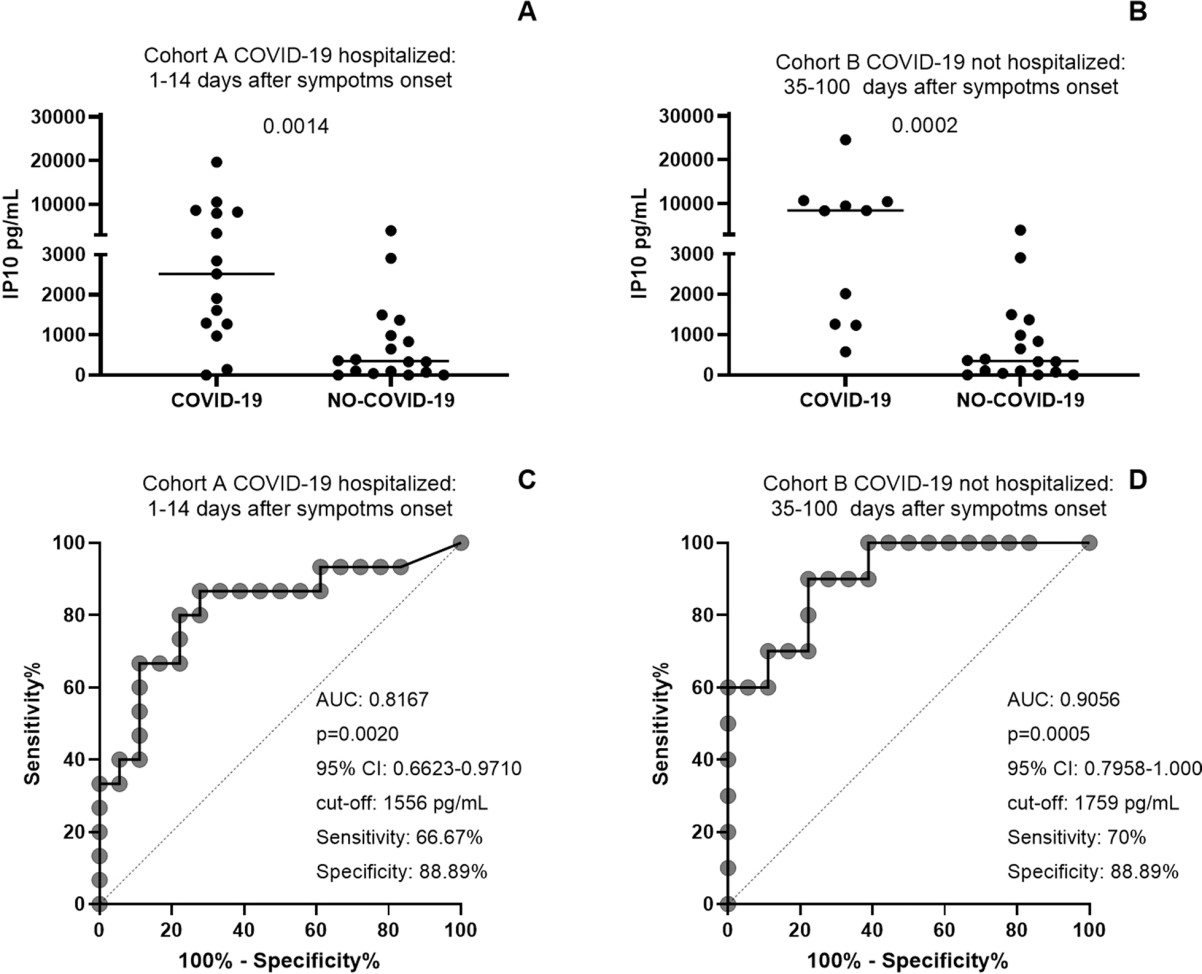
IP-10 modulation in a second cohort of COVID-19 patients. IP-10 production was measured by ELISA in plasma collected after stimulating whole-blood with Spike peptides. **A**, **B** The horizontal lines represent the median of IP-10 production; statistical analysis was performed using the Mann–Whitney test, and p value was considered significant when  ≤  0.05. **C**, **D** The graphs represent the AUCs obtained by the ROC analysis comparing the NO-COVID-19 subjects with three cohorts of COVID-19 patients. **A**, **C** Hospitalized COVID-19 patients enrolled 1–14 days after symptoms onset. **B**, **D** Not-hospitalized COVID-19 patients enrolled 35–100 days after symptoms onset. *IP* interferon-γ inducible protein; *CI* confidence interval; *AUC* area under the curve

We demonstrated that Spike IP-10 had the highest AUC (0.85, p  <  0.0001; Fig. 4) and that the clinical characteristics of the COVID-19-patients did not affect IP-10 production (Table 3). Based on these results, we further evaluated the production of IP-10 in a new study population of NO-COVID-19 and COVID-19-patients stratified according to the hospitalization status and symptoms onset (Table 4). To verify the consistency of our findings, we used a different experimental setting: IP-10 was detected using a routine approach as the enzyme-linked immunosorbent assay (ELISA) and Spike peptides were obtained from a commercial source (Miltenyi). IP-10 production significantly increased after Spike stimulation in the cohort A of “hospitalized COVID-19-patients enrolled between 1 and 14 days after symptoms onset” (p  =  0.0014) and in the cohort B of “not hospitalized COVID-19-patients” (p  =  0.0002), (Fig. 5A–B). ROC analysis demonstrated a high and significant AUC in cohort A and cohort B (AUC: 0.8167; p  =  0.0020; AUC: 0.9056; p  =  0.0005) (Fig. 5C–D). The specificity of the test to identify COVID-19 was 88.89% for both COVID-19-cohorts; the sensitivity was 66.67% for cohort A and 70% for cohort B (Fig. 5C–D).

**Table 4:** Demographical and clinical characteristics of the enrolled subjects for the IP-10 study

	COVID-19	COVID-19	NO-COVID-19	p value
	Cohort A	Cohort B	N = 18	
	N = 15	N = 10		
Hospitalized N (%)	15 (100)	0 (0)	0 (0)	–
Enrolled “X” days after symptoms onset	14-Jan	35–100	/	–
Age median (IQR)	63 (52–70)	55 (31–60)	44 (38–53)	< 0.0001*
Male N (%)	11 (73)	0 (0)	13 (68)	0.0008^§^
Origin N (%)				
West Europe	15 (100)	9 (90)	18 (100)	0.208^§^
East Europe	0 (0)	1 (10)	0	
Asia	0 (0)	0 (0)	0	
Swab positive results N (%)	14 (93)	5 (50)	0 (0)	0.0036^§§^
Serology results IgM N (%)^a^				
IgM +	10 (66.7)	2 (22.2)	0 (0)	
IgM −	4 (26.6)	5 (55.6)	0 (0)	0.2999^§§^
IgM doubtful	1 (6.7)	2 (22.2)	0 (0)	
Serology results IgG N (%)^a^				
IgG +	12 (80)	8 (88.9)	0 (0)	
IgG −	2 (13.3)	1 (11.1)	0 (0)	0.820^§§^
IgG doubtful	1 (6.7)	0 (0)	0 (0)	
Severity N (%)#				
Mild	0 (0)	9 (90)	/	
Moderate	4 (26.7)	0 (0)	/	< 0.0001^§§^
Severe	8 (53.3)	1 (10)	/	
Critical	3 (20)	0 (0)	/	

Bold values indicate *p* values<0.05,

*COVID-19* COronaVIrus Disease 19; *N* number

^a^Missing information for one not hospitalized patients, the percentage has been calculated on 9 patients

^*^Mann Whitney test

^§^Chi-square test

^§§^Chi-square test performed only on COVID-19 cohorts A vs B

^#^WHO criteria [1]

## Discussion

In this study, by a multivariate exploratory analysis we found the best antigen and the best biomarker to distinguish COVID-19- and NO-COVID-19-individuals. To achieve our goal, we used a whole-blood-platform [10] with a luminex read-out. By the sPLS-DA, we identified a COVID-19 signature based on six immune factors. Our results showed that Spike IFN-γ, Spike IP-10, Spike IL-2; NP Pool1 RANTES; NP Pool2 IP-10 and ORF3a IL-2 are the most important in vitro conditions to distinguish COVID-19- from NO-COVID-19-subjects over all the antigen stimulations. Although we demonstrated that a combined test including all the immune factors reached the best AUC to identify COVID-19 and NO-COVID-19-individuals, we found that the single test based on Spike IP-10 could be a potential new biomarker assay of SARS-CoV-2 infection. Moreover, we validated the use of IP-10 as biomarker of SARS-CoV-2 infection in another cohort of COVID-19-patients with different clinical characteristics. In fact, to corroborate the reproducibility of our results, we performed a validation study testing Spike peptides from a commercial company and using a more feasible routine approach such the IP-10 ELISA. We demonstrated that IP-10 had a good accuracy to identify hospitalized COVID-19-patients in the first two weeks after symptoms onset and not-hospitalized-patients enrolled 35–100 days after symptoms onset.

IP-10 is a chemokine mainly secreted by monocytes, fibroblasts and endothelial cells in response to IFN-*γ* that attracts activated T-cells to foci of inflammation [25]; it has already been described as a potential biomarker for other infectious disease, such as tuberculosis and HCV [26–30] and may be easily measured in condition of immune-depression [30]. In acute COVID-19-patients, IP-10 production is a promising surrogate marker of impaired immune responses [13]. In our study IP-10 production induced by Spike stimulation was the only parameter not affected by any clinical characteristics. We reported that IP-10 identified SARS-CoV-2 infection in the acute phase of disease and in COVID-19-recovered subjects. This result has a double scientific implication. Firstly, it supports the specificity of the immune response to viral-peptides in different clinical conditions; secondly, it suggests a possible application of the “IP-10 and Spike whole-blood test” as a potential additional tool for diagnostic and immune response evaluation of COVID-19-patients during the acute phase of the disease. These findings are in agreement with other cytokine release-based tests applied for the diagnosis of several infectious diseases [31–34]. Moreover, an additional possible application of this whole-blood based cytokine assay is the evaluation of immune response in SARS-CoV-2 vaccine trials. In this context, the IP-10 detection may define the immunogenicity of a Spike-based vaccine, whereas the immune response to the virus infection may be evaluated detecting other factors as RANTES induced by NP.

Previous reports focused on the pre-existing immune response to SARS-CoV-2 in the general population, demonstrating that ORF1-specific T-cells were detected in SARS-CoV-2 unexposed donors [19, 35]. Differently, in recovered COVID-19-subjects, the T-cells mainly recognized the structural proteins [19]. In our study, we observed few modulations of immune factors among COVID-19 and NO-COVID-19 individuals in response to the peptides of accessory protein ORF3a; these data indicate that both groups have a similar immune response and suggest a minor contribution of ORF3a in the immune-specific response in acute-hospitalized COVID-19-patients. In line with previous evidence, the majority of immune modulations concerned to stimulations with structural proteins such as NP and Spike. As already reported [10, 16, 17] we observed a production of both inflammatory and anti-inflammatory cytokines and chemokines in response to the structural protein of SARS-CoV-2.

More than 90% of seroconverters COVID-19-individuals shows an immunological memory of T-cell compartment [36] and antibody response, for several months after infection [36, 37]. However, we need more longitudinal studies to understand exactly if the immune memory response remains stable over time. Considering that the early prediction of disease progression could be useful to assess the optimal treatment strategies, an integrated knowledge of the T-cell and antibody response lays the foundation to develop biomarkers to monitor the course of COVID-19 disease.

The limits of the present study are related to the low amount of patients evaluated. However, five different viral antigens and 27 markers were concomitantly evaluated and validated in different cohorts making the here generated evidence robust. Moreover, in the control group of NO-COVID-19 individuals, it would have been useful to include subjects with acute respiratory diseases, as Influenza. Indeed, it has been demonstrated that serum or plasma IP-10 is increased in several respiratory infections, as tuberculosis [26, 38] or influenza [39]. However, in 2020 and 2021 so far, in Europe the Influenza Virus positivity in sentinel specimens remained below the epidemic threshold due to the use of massive vaccination, masks and lockdown rules [40]. Further studies will help understanding if the coinfection of COVID-19 and other acute infectious diseases may have an impact of the SARS-CoV-2-specific IP-10 signature. Nevertheless, in a recent study [10] we showed that NO-COVID-19 patients with respiratory disease such as tuberculosis and bacterial pneumonia did not show IFN-γ-specific response to Spike stimulation. Similarly, in the present study, we did not find IP-10-specific response to Spike in NO-COVID-19 individuals. Interestingly, the NO-COVID-19 group included seven subjects with active tuberculosis under therapy and 5/7 in the acute phase of the disease as they were enrolled within 7 days of diagnosis and of starting the anti-TB specific therapy. These evidences support the specificity of our data even if generated with a low number of control patients.

In conclusion, we demonstrated the potential application of a whole-blood based platform that allowed the selection of the best antigen and best read out to evaluate the immune response to SARS-CoV-2 infection. We also identified IP-10 detection induced by Spike stimulation, as a good in vitro setting to distinguish COVID-19 from NO-COVID-19-individuals.

## Materials and methods

### Study design

This study was approved by the Ethical Committee of Lazzaro Spallanzani National Institute of Infectious Diseases (59/2020) and was conducted between July 15th and November 5th, 2020. Informed, written consent was required to prospectively enroll patients and controls by physicians. Demographic and clinical information were collected at enrollment (Table 1). The study complied with the principles of the Declaration of Helsinki. Inclusion criteria for COVID-19-patients: a diagnosis based on positive nasopharyngeal swab for SARS-CoV-2; a disease with specific clinical characteristics [41]. Exclusion criteria: HIV infection, inability to sign an informed consent and age younger than 18 years. To perform the multiplex analysis, we prospectively enrolled 23 COVID-19-patients and 18 individuals without COVID-19 (NO-COVID-19). COVID-19-patients were classified as asymptomatic (n  =  2), mild (n  =  3), moderate (n  =  11), severe (n  =  5), and critical (n  =  2) (1). NO-COVID-19-individuals were healthy donors (n  =  4), subjects with tuberculosis under therapy (n  =  7) (5/7 were enrolled within 7 days of starting a specific anti-tuberculosis therapy), and subjects with latent tuberculosis infection (n  =  7).

For the IP-10 study, we prospectively enrolled 18 NO-COVID-19-subjects and two cohorts of COVID-19-patients: cohort (A) 15 hospitalized-patients enrolled 1–14 days after symptoms onset; cohort (B) 10 not-hospitalized-patients (convalescent/recovered) enrolled 35–100 days after symptoms onset (Table 4).

### Peptide pools and stimuli

For the exploratory study, SARS-CoV-2 peptide pools of 15-mers (55 peptides) at 2 µg/mL, covering the whole NP (Pool1 and Pool 2), M, ORF3a proteins and 40.5% of the Spike protein, were used as reported [42]. For the validation study, SARS‑CoV-2 PepTivator^®^ Peptide Pool of the Spike protein at 0.1 µg/mL (Miltenyi, Biotec, Germany) were used. Stimulated whole-blood was overnight incubated at 37 °C, 5% CO_2_, plasma was collected and stored at  − 80 °C until used.

### SARS-CoV-2 serology

SARS-CoV-2 specific IgM and IgG levels were measured by ELISA according to manufacturer’s instructions (DIESSE Diagnostica Senese S.p.a., Monteriggioni, Italy). The ratio between the optical density (OD) of the sample and that one of the cut-off reagent (index) was calculated. The samples were scored positive (index  >  1.1), doubtful (index between 1.1 and 0.9) and negative (index  <  0.9).

### Cytokines, chemokines and growth factors evaluation

Bio-Plex Pro Human Cytokine 27-plex Assay panel and the MagPix system (Bio-Rad, Hercules, CA, USA) were used to evaluate in harvested plasma: cytokines, chemokines and growth factors (IL-1β, IL-1RA, IL-2, IL-4, IL-5, IL-6, IL-7, IL-8, IL-9, IL-10, IL-12p70, IL-13, IL- 15, IL-17A, eotaxin, FGF, granulocyte-colony stimulating factor [G-CSF], granulocyte macrophage colony-stimulating factor [GM-CSF], IFN-γ, IP-10, monocyte chemoattractant protein-1 [MCP-1], macrophage inflammatory protein [MIP]-1α, MIP-1β, Platelet-derived growth factor [PDGF], RANTES, tumour necrosis factor-alpha [TNF-α], and vascular endothelial growth factor [VEGF]). Raw data were generated using the Bio-Plex manager software. Concentrations below the detection range were considered as zero. Concentrations above the detection range were converted to the highest value of the standard curve. Analyte levels were subtracted from the unstimulated control. Values generated from less than 50 beads reading were not calculated (one value was missing for: IL-2 ORF3a, IL-5 ORF3a; IL-12 ORF3a; IL-13 ORF3a; IL-15 ORF3a; IL-17 ORF3a; IL-17 NP Pool1; eotaxin Membrane; eotaxin Spike; GM-CSF NP Pool1; GM-CSF NP Pool2; GM-CSF ORF3a; GM-CSF Membrane; MIP-1 α ORF3a; TNF-a NP Pool1; VEGF NP Pool1. Two values were missing for TNF- α ORF3a; VEGF NP Pool 2; VEGF Spike. Three values were missing for VEGF-b ORF3a).

In the validation study, IP-10 was measured in plasma using Human CXCL10/IP-10 Quantikine ELISA (R&D Systems, Abingdon, UK) according to the manufacturer’s instructions. The samples were tested as diluted 1:50. The concentration range of detection was: 7.8–500 pg/mL.

### Statistical analysis

Data were analysed using Graph Pad (GraphPad Prism 8 XML ProjecT), Stata (Stata 15, StataCorp. 2017. Stata Statistical Software: Release 15. College Station, TX: StataCorp LLC) and R Project Software (version 3.6.1). Medians and interquartile ranges (IQRs) were calculated. Mann Whitney U test for comparisons among groups; Chi-squared test for categorical variables; receiver-operator characteristic (ROC) analysis for evaluating the area under the curve (AUC) and the diagnostic performance; Spearman Rank Correlation to measure the strength of association between two variables and the direction of the relationship (positive or negative).

We performed a multivariate exploratory analysis, sparse partial least squares discriminant analysis (sPLS-DA), to identify the most important soluble factors discriminating COVID-19- from NO-COVID-19-individuals*.* The sPLS-DA performed a variables reduction, generating latent components to synthetize the data information*.* For the sPLS-DA analysis, we considered in the model all the 135 analytes simultaneously (5 different stimuli, 27-factors each) limiting the components construction to the first 20 most important variables identified by the method. Data were analyzed with the R-package MixOmics. We performed a logistic regression analysis to evaluate the potential ability of a minimal subset of variables to classify COVID-19 from NO-COVID-19-patients; AUC and p values were reported.

## Data Availability

The data sets generated during and/or analyzed during the current study are available in our institutional repository after request at rawdata.inmi.it.
